# Overcoming the Low Bioavailability of Apigenin: The Therapeutic Efficacy for Androgenetic Alopecia Through Topical Administration

**DOI:** 10.1002/fsn3.71155

**Published:** 2025-11-27

**Authors:** Yanxi Peng, Huan Zhou, Yiqing Sun, Dane Huang, Chao Zhao

**Affiliations:** ^1^ School of Public Health Xiangnan University Chenzhou Hunan China; ^2^ School of Basic Medical Sciences Xiangnan University Chenzhou Hunan China; ^3^ State Key Laboratory of Anti‐Infective Drug Discovery and Development, School of Pharmaceutical Sciences Sun Yat‐Sen University Guangzhou China; ^4^ Guangdong Provincial Key Laboratory of Research and Development in Traditional Chinese Medicine Guangzhou China; ^5^ Guangdong Provincial Second Hospital of Traditional Chinese Medicine (Guangdong Provincial Engineering Technology Research Institute of Traditional Chinese Medicine) Guangzhou China; ^6^ The Fifth Clinical College of Guangzhou University of Chinese Medicine Guangzhou University of Chinese Medicine Guangzhou China; ^7^ College of Pharmacology Xiangnan University Chenzhou Hunan China; ^8^ Research and Development Department, Cinaneo Pharmaceuticals, Ltd. Shenzhen China

**Keywords:** AGA, apigenin, CDK5, topical administration

## Abstract

Numerous common foods are abundant in apigenin. These include parsley, celery, chamomile, and snow lotus. Apigenin demonstrates a broad spectrum of biological activities, such as anti‐inflammatory, antioxidant, anti‐cancer, neuroprotective, and antidiabetic effects. Consequently, many scientists have endeavored to develop it as a drug. Nevertheless, there are no commercially available or clinically used drugs containing apigenin, primarily because of its poor water and fat solubility. In this study, a topical administration approach of apigenin was employed to treat skin diseases, aiming to overcome the issue of low bioavailability. Based on the target prediction results, the top 10 targets suggest that androgenetic alopecia (AGA) is a potential target disease. After 28 days of treatment in testosterone‐induced AGA mice, the hair growth status was comparable to that in the minoxidil group and the normal group, and was significantly better than that in the model group. The mRNA level expression in the serum indicated that apigenin might promote hair growth through the Wnt signaling pathway. The docking study revealed that CDK5 is the most likely target of apigenin for treating AGA. This study verified that topical administration of apigenin is a viable strategy for drug development.

## Graphical abstract

This study investigated the application of apigenin for the treatment of androgenetic alopecia (AGA) through topical administration, addressing challenges related to the compound's solubility and bioavailability. After 28 days, mice with testosterone‐induced AGA exhibited hair growth comparable to that observed in the minoxidil treatment group and significantly greater than that in the untreated control group. The research suggested that apigenin may promote hair growth via the Wnt signaling pathway. Further investigation identified CDK5 as a likely target. These findings indicate that topical apigenin could represent a promising avenue for novel treatments for AGA, although it is not currently available as a commercial drug.

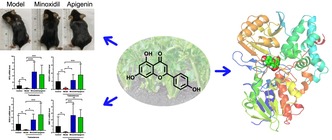

## 1 Introduction

Flavonoids are a vast group of polyphenolic compounds present in fruits, vegetables, and medicinal plants. Among these, apigenin (4′,5,7‐trihydroxyflavone) is notable for its wide distribution and remarkable biological activities (Yan et al. 2017). This flavonoid is commonly found in parsley, celery, chamomile, snow lotus and various other edible plants, making it easily accessible to the general population (Thomas et al. 2023).

Apigenin exhibits a wide range of biological activity including anti‐inflammatory, antioxidant, anti‐cancer, neuroprotective and antidiabetic effects (Yoon et al. 2023). It exerts anti‐inflammatory responses by downregulating transcription factors such as active protein 1 (AP‐1), NF‐κB, and signal transducer and activator of transcription (STAT) (Yoon et al. 2023). The mechanism of its antioxidant activity involves scavenging free radicals and protecting cells from oxidative stress (Kashyap et al. 2022; Shen et al. 2024). Apigenin can also induce apoptosis, inhibit tumor cell proliferation and angiogenesis for treating cancer (Imran et al. 2020). It has been demonstrated to own efficacy against breast, prostate, colon cancer, skin cancer and etc. A previous study also reported that apigenin can be applied to Alzheimer's disease through preventing Aβ‐42 aggregates and tau filaments (Siddique et al. 2022). Apigenin and its derivatives can be employed for diabetes as α‐glucosidase inhibitors (Zeng et al. 2016).

Another advantage of apigenin is its safety. Even when administered at high dosages, no instances of toxicity have been observed (Lotfi and Rassouli 2024). This flavonoid, which is abundantly present in several food sources and consumed in substantial amounts daily, has a proven safety record. However, its poor intestinal absorption, due to its low water solubility, restricts its efficacy and the feasibility of its application as a drug (Borges et al. 2022).

A lot of effort has been dedicated to enhancing the bioavailability of apigenin. One approach involves synthesizing apigenin derivatives to improve their water or lipid solubility, such as adding glucose (Liu et al. 2024). Nanotechnology offers another avenue to enhance the body's absorption or enable precise release (Kumar et al. 2024). The third strategy is to apply apigenin as a topical medicine for the treatment of skin diseases, including androgenetic alopecia (AGA), psoriasis, acne and atopic dermatitis. In this study we aim to investigate the possibility of the medical application of apigenin through topical administration.

AGA, referred to as male‐pattern baldness in men and female‐pattern hair loss in women, is a prevalent dermatological disorder that significantly affects the quality of life of millions globally (Wu et al. 2024). Characterized by a progressive reduction in hair follicle size, a shortened anagen (growth) phase, and an increase in the telogen (resting) phase, AGA causes the gradual replacement of terminal hairs with vellus hairs, eventually leading to visible hair thinning and baldness. The pathogenesis of AGA is multifactorial. Androgens, especially dihydrotestosterone (DHT), play a crucial role. DHT binds to androgen receptors in hair follicles, initiating a series of molecular events that result in follicular miniaturization. Moreover, chronic inflammation, oxidative stress, and genetic predisposition contribute to the development and progression of this condition.

Current therapeutic options for AGA include topical minoxidil, oral finasteride (for men), and low‐level laser therapy. While these treatments can be effective in some cases, they are often associated with limitations such as variable response rates, potential side effects (e.g., sexual dysfunction with finasteride), and the need for long‐term administration (Luo et al. 2024). As a result, there is a growing interest in identifying natural compounds from food with potential anti‐alopecia properties. Apigenin, as a food extract, possesses potential therapeutic activity against hair loss, precisely meeting this demand.

## 2 Methods

### 2.1 Reagents

The concentration of apigenin solution for in vivo study is 0.4 mg/mL. It was dissolved in a solution of mixed white oil and water at a ratio of 14: 1, and then an apigenin emulsion was prepared by ultrasonic treatment for 0.5 h. The testosterone solution was prepared by dissolving testosterone into 75% ethanol with a final concentration of 0.05%.

### 2.2 Establishment of a Mouse Model of AGA Animal Experiment

For the welfare of experimental animals, the animal study described in this manuscript was approved by the Institutional Animal Care and Use Committee of Guangdong Provincial Second Hospital of Traditional Chinese Medicine (Guangdong Provincial Engineering Technology Research Institute of Traditional Chinese Medicine) under approval number 049109. All procedures strictly adhered to the guidelines for the care and use of laboratory animals. A total of 32 C57BL/6 mice were applied in this study. Following a 1‐week acclimatization period, the dorsal skin of the mice was shaved with an electric clipper, followed by depilation with a cream. Mice without visible wounds on their backs were selected and randomly assigned to groups: Control, Model, Minoxidil, and Apigenin groups. There were eight mice in each group. Subsequently, a testosterone solution (0.1 mL/cm^2^) was topically applied to the hair loss areas of the Model, Minoxidil, and Apigenin groups once a day for 28 consecutive days. After 0.5 h, the Control, Model and Minoxidil groups were treated with a white oil and water mixture (14:1) applied to the backs daily (0.1 mL/cm^2^), while the Minoxidil group received 5% Minoxidil (0.1 mL/cm^2^) (Aldhalimi et al. 2014), and the Apigenin group was treated with apigenin (0.4 mL/cm^2^, dissolved in clean water) (Li et al. 2023). Daily observation and scoring of hair growth in the affected regions on the backs of the mice were conducted.

### 2.3 Quantitative Real‐Time PCR (RT‐qPCR)

The mice were humanely euthanized, and the back skin tissue was collected. The samples were immediately frozen in liquid nitrogen and stored at −80°C for later analysis. Following the manufacturer's protocol, total RNA was extracted from the preserved tissue. For mRNA expression level analysis, 0.25 μg total RNA was reverse‐transcribed into cDNA by using the Evo M‐MLV RT Premix for the next qPCR (Accurate Biology, China). DNA amplification was performed using the SYBR Green Premix Pro Taq HS qPCR Kit (Accurate Biology, China) with 0.5 μL of ROX reference Dye (Accurate Biology, China) and was conducted in a StepOnePlus Real‐Time PCR system (Thermo Fisher Scientific, USA) using SYBR Green detection chemistry with the resulting cDNAs. Primer sequences used are shown below:

**Table:** 

		Sequences (5′—3′)
IGF‐1	Forward	GAGGGGCTTTTACTTCAACAAG
	Reverse	TACATCTCCAGTCTCCTCAGAT
BMP‐2	Forward	AGTAGTTTCCAGCACCGAATTA
	Reverse	CACTAACCTGGTGTCCAATAGT
GSK‐3β	Forward	TGGTAGCATGAAAGTTAGCAGA
	Reverse	CTCTCGGTTCTTAAATCGCTTG
SOX‐2	Forward	ATGAAGGAGCACCCGGATTATA
	Reverse	GGAAGCGTGTACTTATCCTTCT
VEGFA	Forward	GGGCTCTTCTCGCTCCGTAGTAG
	Reverse	CCCTCTCCTCTTCCTTCTCTTCCTC
HGF	Forward	ACCTACAGGAAAACTACTGTCG
	Reverse	TGCATTCAACTTCTGAACACTG

### 2.4 Docking

For the docking studies, Molecular Operating Environment 2014.10 (MOE, Chemical Computing Group Inc. Montreal, Canada) was used, operating on Windows 11 on a DELL computer (Intel i5, 2.8 GHz CPU, 8GB memory). The structures of apigenin were drawn in the MOE package with standard bond lengths and angles, and minimized using the conjugate gradient method. The Gasteiger‐Huckel charge was applied for the minimization process, with a distance‐dependent dielectric function. A preliminary docking study was carried out using the crystal structure of CDK1 (PDB code: 5LQF), CDK5 (PDB code: 4 AU8), CYP19A1 (PDB code: 3S79), ER‐α (PDB code: 1XP1), AchE (PDB code: 4EY7). The structure was polished as follows: (1) all water molecules were removed from the crystal structure; (2) the QuickPrep module was applied. The docking study was performed with all compounds in a maximum of 30 poses through rigid receptor refinement. For NOX4, the crystal structure was obtained from AlphaFold (ID: AF‐Q9NPH5‐F1‐v4). This structure was superposed with NOX5 (PDB code: 5O0T), and then the NOX5 structure was removed. The HEM in 5O0T was selected as a ligand for docking.

### 2.5 Statistical Analysis

All statistical analyses were performed using GraphPad Prism Version 9. Results are presented as the mean ± SEM. In vitro studies represent biological replicates, while in animal studies, *n* represents the number of mice. For multi‐group comparisons, a one‐way ANOVA was used to compare group differences, followed by an uncorrected Fisher's LSD test. A *p* value < 0.05 was considered statistically significant.

## 3 Results

### 3.1 Apigenin Exhibited Potential Anti‐AGA Activity

Dysregulation of the inflammatory equilibrium is the primary reason for skin immunological diseases, including AGA, psoriasis, acne, atopic dermatitis. Therefore, apigenin may be applied for skin disease treatment due to its potent anti‐inflammatory efficiency. To investigate its activity, we firstly predict the relationship between potential targets of apigenin and skin disease. In this study, AGA, psoriasis, acne and atopic dermatitis were selected since they are the most common skin diseases. The SwissTargetPrediction (http://www.swisstargetprediction.ch/) was performed to predict the potential targets of apigenin, and the top 10 targets were listed in Table 1. Except for Aldo‐keto reductase family 1, member B1 (AKR1B1) and Xanthine dehydrogenase (XDH), all the others are related to skin disease. Moreover, six of them (NADPH oxidase 4 (NOX4) (Jeon et al. 2023), cyclin‐dependent kinase 5 (CDK5) (Zhai et al. 2018), cytochrome P450 19A1 (CYP19A1) (Li et al. 2024), estrogen receptor α (ER‐α) (Moverare, Lindberg et al. 2002), CDK1 (Łukasik et al. 2022) and acetylcholinesterase (AchE) (Leem et al. 2022)) are potential therapeutical targets of AGA. Similarly, NOX4 (Zheng et al. 2019), CDK5 (Staniszewska et al. 2023), FLT3 (Zhu et al. 2021), ER‐α (Iwano et al. 2020), and CDK1 (Li et al. 2021) are targets for psoriasis treatment. Acne and atopic dermatitis were three targets related. Hence, AGA was selected for further study.

**TABLE 1:** The relationship between predicted targets of apigenin and skin diseases.

No.	SwissTargetPrediction	AGA	Psoriasis	Acne	Atopic dermatitis
1	NOX4	Y	Y	N	N
2	AKR1B1	N	N	N	N
3	CDK5	Y	Y	N	N
4	XDH	N	N	N	N
5	MAOA	N	N	Y	N
6	FLT3	N	Y	N	N
7	CYP19A1	Y	N	Y	N
8	ER‐α	Y	Y	Y	Y
9	CDK1	Y	Y	N	N
10	ACHE	Y	N	N	Y

### 3.2 Apigenin Promotes Hair Growth in AGA Mice Model

**FIGURE 1:**
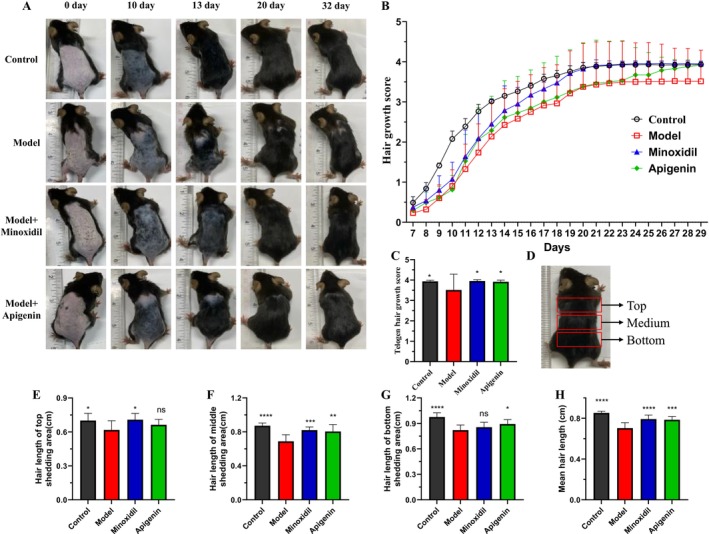
Apigenin increased hair growth in AGA mice. (A) Representative optical images of hair growth over increasing days. (B) The hair growth score over increasing days; the score is calculated according to back skin covered by regrown hairs. (C) The telogen hair growth score. (D) Schematic diagram of the zones of hair regeneration on the back of the animal. (E) The hair length of the top shedding area. (F) The hair length of the middle shedding area. (G) The hair length of the bottom shedding area. (H) The mean hair length of all new hair growth. Statistical difference was determined by ordinary one‐way ANOVA. ^ns^ *p* ≥ 0.05, *p < 0.05; **p < 0.01; ***p < 0.001; ****p < 0.0001.

According to our assumption, the topical administration method was applied to overcome the problem of low bioavailability of apigenin. Therefore, in this study, the testosterone‐induced AGA model was performed to evaluate whether apigenin could effectively promote hair growth. Starting from the 7th day of administration, we scored the hair growth by the proportion of newly grown hair in the model area to evaluate the promoting effect of the drug on hair growth. As shown in Figure 1B, from the 7th to the 12th day, the minoxidil group, the apigenin group and the model group showed the same growth rate. From the 12th to the 23rd day, apigenin did not show a promoting effect on hair growth, which was consistent with the model group. Then, starting from the 24th day, the hair growth in the apigenin group increased rapidly and reached the same level as the minoxidil group and the normal group on the 29th day. The growth score of apigenin was significantly better than that of the model group (Figure 1C). Further, the hair was divided into three groups: top, middle and bottom (Figure 1D), and the length was estimated. Compared with the model group, apigenin could slightly increase the hair length in the upper back, but without significance (Figure 1E). However, apigenin could significantly increase the hair length in the middle and bottom back, and even exceeded the positive drug minoxidil in the bottom back (Figure 1F,G). Compared with the model group, apigenin could significantly increase the length of all newly grown hair. Thus, apigenin can effectively increase the hair length of AGA mice and has a therapeutic effect on hair loss.

### 3.3 Apigenin Regulated the Key Hair Growth Gene Level

**FIGURE 2:**
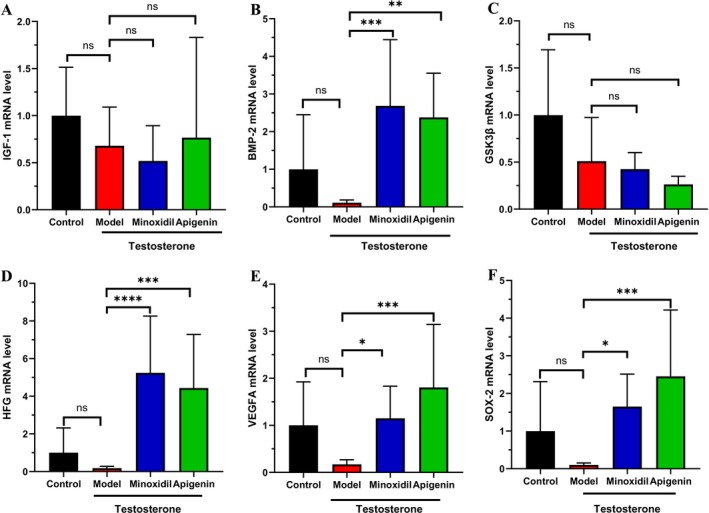
Apigenin increased hair growth gene expression. (A) The serum IGF‐1 mRNA level in AGA mice. (B) The serum BMP‐2 mRNA level in AGA mice. (C) The serum GSK‐3β mRNA level in AGA mice. (D) The serum HFG mRNA level in AGA mice. (E) The serum VEGFA mRNA level in AGA mice. (F) The serum SOX‐2 mRNA level in AGA mice. Statistical difference was determined by ordinary one‐way ANOVA. ^n*s*^ *p* ≥ 0.05, *p < 0.05; **p < 0.01; ***p < 0.001; ****p < 0.0001.

Based on the findings of the in vivo study, apigenin can rapidly accelerate hair growth within 5 days. This indicates that apigenin might regulate the expression of proteins associated with hair growth. Consequently, the key genes related to hair growth were detected. Insulin‐like growth factor 1 (IGF‐1) is of great significance in hair growth and is involved in various types of alopecia. Its stimulation can promote hair growth (Zhao et al. 2020). Bone morphogenetic protein 2 (BMP‐2) is typically downregulated in patients with androgenetic alopecia (AGA). Since androgens can reduce BMP‐2 in dermal papilla cells (DPC), this impairs the DPC's capacity to induce hair follicle stem cell differentiation, potentially resulting in hair loss (Ceruti et al. 2021). Glycogen synthase kinase 3β (GSK‐3β) is a key regulator of the Wnt signaling pathway, which is crucial for hair follicle development and hair cycling. Inhibiting GSK‐3β leads to promoting hair growth. Hepatocyte growth factor (HGF) is secreted by cells in the hair follicle, particularly the DPC, and it stimulates neighboring follicular epithelial cells to promote hair follicle growth (Ki et al. 2020). Vascular endothelial growth factor (VEGF) A can support hair growth by facilitating nutrient supply to the hair follicle and increasing its size (Ding et al. 2024). SRY‐box 2 (SOX‐2), which is expressed in DP cells, regulates the migration of differentiating hair shaft progenitors and finetunes BMP signaling to promote hair growth (Zhou et al. 2024). Apigenin did not regulate the mRNA levels of all hair growth‐related proteins. Among them, IGF‐1 and GSK‐3β were not promoted or decreased in the apigenin‐treated mice respectively (Figure 2A,C). On the other hand, apigenin can significantly increase the BMP‐2, HGF, VEGFA and SOX‐2 mRNA levels (Figure 2B,D–F). Among these, the increase in the expression of VEGFA and SOX‐2 genes is more effective than minoxidil. This result is consistent with the previously observed rapid hair growth.

### 3.4 CDK5 Is the Potential Target of Apigenin for AGA

As mentioned before, SwissTargetPrediction predicted that six targets of apigenin are related to AGA. To further confirm the possibility of these targets, molecular docking was performed. Except for NOX4, the crystal structures of the other proteins in complex with their ligands were utilized for docking. Since NOX4 has no crystal structure, the corresponding structure was downloaded from AlphaFold for docking. According to the results of binding free energy, apigenin has good binding with NOX4, CDK1, ER‐α, CDK5, CYP19A1 and AchE. Among these, CYP19A1 and CDK5 have the lowest binding energy (Table 2).

**TABLE 2:** The free energy of apigenin binding to targets.

Targets	Free energy
NOX4	−6.3924
CDK1	−6.3075
CDK5	−7.3707
CYP19A1	−6.8413
ER‐α	−6.5359
ACHE	−6.6545

**FIGURE 3:**
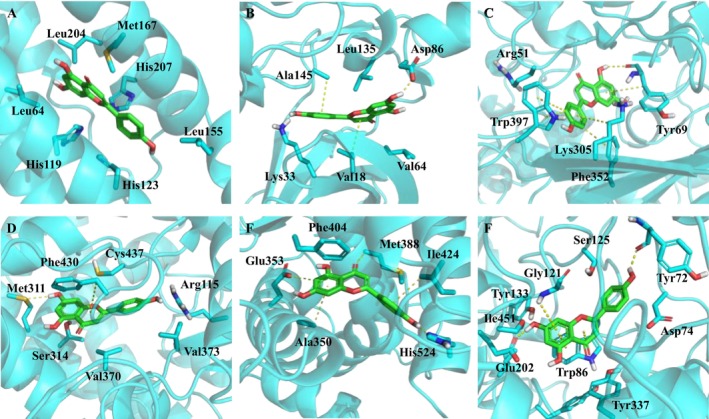
The binding model of apigenin with six potential targets. (A) NOX4. (B) CDK1. (C) CDK5. (D) CYP19A1. (E) ER‐α. (F) AchE. The yellow dashed line represents the interactions of H‐bond or π‐π stacking.

Subsequently, the binding model of apigenin with these targets were analyzed. Apigenin was fit into the pocket composed of Leu204, Met167, His207, Leu155, His123, His119 and Leu64 on NOX4 (Figure 3A). The interaction was mainly achieved through van der Waals forces. The oxygen in the ring was located in the hydrophilic region formed by His207 and His119, while the two benzene rings corresponded to the hydrophobic pocket. Similarly, apigenin formed π‐π stacking interactions with Ala145 and Val18 in CDK1 (Figure 3B), and the hydroxyl group formed a hydrogen bond with the carbonyl group of Asp86. Apigenin interacted with Trp397, Lys305 and Phe352 on CDK5 through four π‐π stacking interactions (Figure 3C). One H‐bond was formed by apigenin and Tyr69. At the same time, it was also located in a lipophilic pocket. The binding mode of apigenin with CYP19A1 showed that Met311 and Cys437 provided a hydrogen bond and π‐π stacking interactions respectively. The phenolic group was located in the hydrophobic region formed by Val379 and Val373, and the hydroxyl group on the phenolic group corresponded to the hydrophilic Arg115, which greatly enhanced the affinity (Figure 3D). Apigenin still interacted with ER through π‐π stacking interactions with Ala350 and Ile424 (Figure 3E). His254, Glu353, and Met 388 provided H‐bonds with apigenin. As shown in Figure 3F, apigenin formed hydrogen bonds with Tyr72 and Glu202 on the AchE protein respectively. At the same time, Gly121 and Trp86 provided π‐π stacking interactions. From this, it can be seen that apigenin has good affinity with the target targets. However, the specific target cannot be determined yet.

According to the gene expression results in the blood, we hypothesized that apigenin promotes hair growth by activating the Wnt pathway. Therefore, further investigations were carried out on whether these six targets are related to the Wnt signaling pathway. Inhibitors of CYP19A1 (Gobbi et al. 2006) and NOX4 can interfere with the activity of the Wnt pathway. The expression of the AchE gene and protein is regulated by the Wnt signaling pathway (Jensen et al. 2012). The role of ER‐α in hair growth is complicated. Both agonists and antagonists work in hair growth (Ohnemus et al. 2005). While, ER‐α antagonist can cause Wnt pathway inhibition through degradation of β‐catenin (Bhat et al. 2021). Hence, it was temporarily excluded from the list of potential targets. Inhibitors of CDK1 and CDK5 can activate the Wnt signaling pathway (Zhao et al. 2025). The free energy and binding model of apigenin with CDK5 is better than CDK1. Therefore, CDK5 is regarded as a potential target for apigenin to promote hair growth.

## 4 Discussion

Apigenin, as a readily accessible flavonoid natural product, possesses a wealth of biological activities, such as anti‐inflammatory, antioxidant, anti‐cancer, antibacterial, antiviral, neuroprotective, and cardio‐cerebrovascular protective effects. Additionally, apigenin has very low toxicity; therefore, scientists are highly interested in developing apigenin into a drug. However, the low bioavailability of apigenin has become a bottleneck issue, and no apigenin‐based drug has yet entered clinical trials. Currently, a mainstream approach is to develop new dosage forms to overcome this problem, but no substantial progress has been made. Thus, topical application seems to be a more favorable solution, yet few have explored this approach. In this study, we attempted to treat androgenetic alopecia by topical application of apigenin and achieved good results, demonstrating that topical apigenin is a potential direction for drug development.

During the experiment, we found that apigenin did not promote hair growth at the beginning. However, within just 5 days from the 24th to the 29th day, the hair growth rate rapidly increased and caught up with minoxidil. This interesting phenomenon might be due to the insufficient dosage at the start. Once the drug accumulation reached the therapeutic level, it demonstrated a very potent effect. The low toxicity of apigenin allows us to administer higher doses for treatment, thereby accelerating hair growth. In the future, we will continue to explore the use of high‐dose administration.

After administration of the drug, the mRNA levels of proteins related to hair growth were all regulated to normal levels. According to these results, apigenin may promote hair growth by activating the Wnt signaling pathway. VEGFA, a protein with the function of activating the Wnt pathway, was significantly increased in the blood of mice after apigenin treatment. At the same time, HGF associated with the activation of the Wnt pathway was also elevated. HGF can activate the Wnt/β‐catenin pathway through the transcriptional activation of LEF1. GSK‐3β, an inhibitor of the Wnt pathway, was also downregulated, although without significant differences. SOX‐2, a protein that has a coordinating effect on the Wnt pathway, was also upregulated. Finally, the mRNA expression of the Wnt target gene BMP‐2 was increased. All the above evidence indicates that the Wnt pathway is an important mechanism for apigenin to promote hair growth.

Finally, based on the results of the in vivo study and molecular docking, CDK5 was initially identified as a potential target of apigenin. In fact, CDK1 is also highly likely to be a target of apigenin. According to the binding free energy, the selectivity of apigenin between CDK5 and CDK1 is not significant, and further experiments are needed to explore this. On the other hand, apigenin is also highly likely to act as an agonist of ER to play a role in hair growth, but the more detailed mechanism still needs to be explored. Previously, no topical drugs targeting these three proteins have been reported, which also provides a new strategy for flavonoid development.

## 5 Conclusion

In this study, topical administration was applied to overcome the problem of apigenin's low bioavailability and successfully demonstrated that apigenin can be used to treat AGA. Based on the rate of hair growth, apigenin may treat AGA through promoting hair growth. Additionally, the expression level of different protein mRNAs in serum confirmed this. Meanwhile, the results of qPCR also suggested that the mechanism of apigenin is activating the Wnt pathway. Finally, combined with the results of molecular docking, CDK5 was identified as a potential target of apigenin.

## Author Contributions

**Yanxi Peng:** data curation, conceptualization (equal), formal analysis, writing – original draft. **Huan Zhou:** data curation. **Yiqing Sun:** data curation. **Dane Huang:** methodology (equal), project administration (equal), conceptualization (equal). **Chao Zhao:** supervision, writing – review and editing.

## Ethics Statement

The authors have nothing to report.

## Consent

The authors have nothing to report.

## Conflicts of Interest

The authors declare no conflicts of interest.

## Data Availability

The data that support the findings of this study are available from the corresponding author upon reasonable request.
